# Numerical study of reflection-based energy density enhancement in turbid media via wavefront shaping

**DOI:** 10.1364/BOE.591736

**Published:** 2026-05-11

**Authors:** Niklas Fritzsche, Manuel Petzi, Dominik Reitzle, Alwin Kienle

**Affiliations:** 1 Institut für Lasertechnologien in der Medizin und Meßtechnik an der Universitäat Ulm, 89081 Ulm, Germany; 2 Faculty of Natural Sciences, Ulm University, D-89081 Ulm, Germany

## Abstract

Delivering energy deep into turbid media is essential for applications like biomedical imaging, medical treatment, and material processing. We applied wavefront shaping to enhance light penetration by manipulating the phase of incident light. Using feedback-based phase optimization and numerical solutions of Maxwell’s equations, we present an approach that significantly and non-invasively increases the energy density deep inside complex scattering media by minimizing the reflected light, without prior knowledge of the media. In simulations of media with a transport mean free path as low as 43.36μm , we achieved a 2.5-fold enhancement of the energy density at a depth of about 200μm . Light transport through this simulated medium is equivalent to propagation through approximately 4mm of biological tissue with optical properties typically found in the red or infrared wavelength range. By switching between minimized and maximized reflection, the energy density can be modulated by up to a factor of 12 deep inside the scattering medium, while the energy density at shallow depths remains fairly unchanged.

## 1. Introduction

Delivering energy deep into turbid media is a fundamental problem in many fields, including various imaging modalities, material processing and communications [1]. An efficient technique for increasing the amount of light transported deep into turbid media is wavefront shaping. First presented by Vellekoop and Mosk [2], this technique manipulates light by adjusting its phase. Hence, light incident on a scattering sample can be redirected to a desired location, increasing the light intensity due to constructive interference at an arbitrary location inside or behind the medium. For the development of efficient optimization algorithms [3–7] that have the potential to provide such an increase in energy density at larger depths, a comprehensive understanding of the effects within the medium that influence the transport is required. In this context, the internal field distribution, and especially the channels with high transmission (open transmission channels), are of primary interest and have been the subject of thorough theoretical [8,9] and experimental [10–12] investigation. These open channels, which incur almost no loss, can be determined via the transmission matrix [13,14], which can then be used to study energy deposition inside random media [15,16]. Significant progress in this area has already been made via time-gating [17–20] and the introduction of a deposition matrix [21]. Further experimental work has been conducted on the enhancement of energy transported into turbid media for on-chip disordered nanostructures [22], energy density probing via the enhancement of light originating from fluorescent nanospheres buried inside a disordered medium [23], and the enhancement of reflection or transmission through thin scattering layers [24].

This work aims to minimize the reflected intensity from turbid media via wavefront shaping, thereby enhancing energy delivery deep into optically thick media. The optimization method presented in this work does not require any prior knowledge of the refractive index distribution, positions of the scattering and absorbing particles, or any pre-calculations, such as determining the reflection or transmission matrix. It also does not involve the determination and targeted use of the sample-specific channels.

Using a two-step beam synthesis method based on numerical solutions of Maxwell’s equations [25], in combination with the aforementioned feedback-based phase optimization, a robust and fundamental methodology is presented that minimizes the total detected reflection while simultaneously increasing the penetration depth of the light coupled into the medium. In addition, a comparison is made between the results for minimized reflection and those for maximized reflection. Investigations into this have already been undertaken by Bender et al. [26].

The simulations solving Maxwell’s equations are conducted to obtain near-field solutions, which can in turn then be transferred to a far-field distribution which resembles the signal that is to be optimized. Furthermore, they enable direct access to the internal energy density distribution, and thus allow to analyze the optimization process, depending on simulation parameters and the properties of the investigated media. The optimization procedure is designed to be straight forwardly transferable to possible experiments, where the simulations conducted to obtain the near-field solutions can be replaced by measurements on real scattering systems, like biological tissue.

The investigated media have a transport mean free path as low as $\ell^{\prime }\approx 43.36\;\mu \text{m}$ and an optical thickness of $L/\ell^{\prime }\approx 4.4$ , where L is the thickness of the simulated area. The results obtained show that this technique is an excellent approach for improving methodologies that rely heavily on the amount of light deep inside the investigated scattering medium, as is, for example, the case for many therapeutic treatments.

## 2. Simulations

### 2.1. Two-step beam synthesis method for solving Maxwell’s equations

Our simulations aim to modify the energy density inside a medium by minimizing the reflected far-field intensity via phase optimization. Since interference effects play a central role, it is necessary to work on the level of fields using Maxwell’s equations, rather than on the level of intensities, by applying, for example, Monte Carlo simulations to solve the radiative transfer equation. To solve the inhomogeneous Maxwell’s equations numerically, we employ a Born series approach [27,28]. The algorithm and implementation of our solver have been described in detail by Krueger et al. [28]. The computational domain is a two-dimensional rectangular region in the x-y plane, and the input to the solver is a position-dependent complex refractive index. The solver assumes out-of-plane polarization of the electric field (i.e., $H_{x}$ , $H_{y}$ , $E_{z}$ ). The truncation of the iteration depends on the difference of the fields in two consecutive iterations. Hereby, the absolute error is the maximum of the absolute value of the difference over all grid points; the relative error is obtained by dividing the absolute error by the absolute value of the previous fields. As truncation conditions, we used an absolute error of $10^{-2}$ , and a relative error of $10^{-3}$ .

We simulate two scattering media with thicknesses of L=100μm and L=192μm along the x direction. The scatterers are non-overlapping discs of radius 1μm and refractive index $n_{\mathrm{disc}}=1.33$ . In the absorbing configurations, the refractive index is modified to 1.33+0.001i (thin medium) and 1.33+0.0001i (thick medium). The discs occupy 10% of each medium’s area, and the surrounding environment has a refractive index of $n_{\mathrm{env}}=1$ .

Biological tissue typically has a reduced scattering coefficient in the order of $\mu_{s}^{\prime }\approx 1\;{\text{mm}}^{-1}$ in the red and infrared wavelength range. Therefore, in the framework of radiative transfer, the simulated medium thickness of 200 µm corresponds roughly to biological tissue with a thickness of about 4 mm, if the reduced scattering coefficient is scaled accordingly. This scaling does not take the actual scatterer sizes and shapes into account. The different scattering phase functions however do not pose a significant problem, because the simulated medium is highly scattering, and light propagation deep in the medium is in the diffuse regime.

We use disc scatterers, because modeling fine structures like cells in tissue requires very high spatial resolution and therefore great amounts of storage and computation time. Moreover, simulations in three spatial dimensions are even more demanding. Additionally, our wavefront shaping procedure requires a large number of incident waves to obtain useful results. Thus, we restrict the computational domain to two dimensions.

**Fig. 1.**
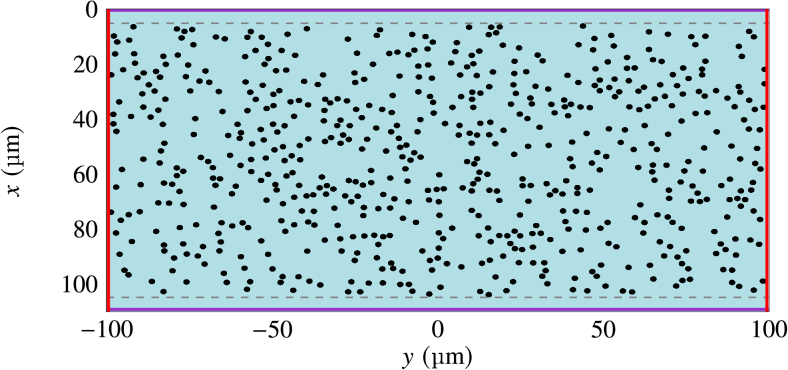
Schematic representation of the thin simulated medium. The scattering discs are embedded in air. Grey dashed lines denote the medium’s front and back surface, purple lines indicate perfectly matched layers, and red lines mark the periodic boundaries.

To suppress reflections at the front and back of the grid, perfectly matched layers are applied above and below the media. Outgoing waves disappear at these boundaries as if they were propagating unobstructed towards infinity. Periodic boundary conditions at y=±100μm (thin medium) and y=±220μm (thick medium), simulate an infinitely extended medium along the y direction. Waves that leave the computational domain on one side re-enter on the opposite one, and therefore there are no reflections at these boundaries. Furthermore, illumination from the side of the computational domain cannot occur, because the periodic boundary conditions ensure that incident plane waves always enter the medium at the front surface. Figure 1 shows a representation of the thin medium in two dimensions. The periodic boundaries, depicted in red, are shown on the left and right sides of the disc distribution displayed. The perfectly matched layers are shown in purple, and the medium’s surfaces are depicted as grey dashed lines.

A spatial grid resolution of $\Delta x=\Delta y=\lambda_{\min}/20$ is chosen for the thin medium, and $\Delta x=\Delta y=\lambda_{\min}/4$ for the thick medium. Although the coarser resolution in the thicker medium introduces minor errors in the dispersion relation [29], it has a negligible impact on the averaged energy density and significantly reduces computational cost. For a vacuum wavelength $\lambda_{0}=1\;\mu \text{m}$ , the minimum wavelength inside the scatterers is $\lambda_{\min}=\lambda_{0}/n_{\mathrm{disc}}\approx 751.88\;\text{nm}$ and Δx=Δy≈37.59nm for the thin medium, as well as Δx=Δy≈187.97nm for the thick medium. Hence, for a grid with $N_{x}=2927$ and $N_{y}=5321$ points in the x and y directions, the computational domain of the thin medium has a size of $L_{x}=110\;\mu \text{m}$ and $L_{y}=200\;\mu \text{m}$ . For the thick medium $N_{x}=1065$ and $N_{y}=2341$ , resulting in a computational domain of $L_{x}=200\;\mu \text{m}$ and $L_{y}=440\;\mu \text{m}$ .

A light beam is synthesized by solving Maxwell’s equations for plane waves incident from several angles, i.e., assembling an angular spectrum of plane waves (ASPW), and taking the sum of the appropriately weighted near-fields. Since the fields have to satisfy periodic boundary conditions, the admissible wave vectors $\vec{k}={\left(k_{x},k_{y}\right)}^{T}$ are determined by the conditions

(1) $$k_{y}L_{y}=2\pi m\;\text{with}\;m\in Z,$$

(2) $$k_{x}=\sqrt{{\left|\vec{k}\right|}^{2}-k_{y}^{2}}$$

for plane waves propagating in the positive x direction. This yields 401 plane waves with a spacing of $\Delta k_{y}=31410.02\;{\text{m}}^{-1}$ (thin medium) and 881 plane waves with $\Delta k_{y}=14278.75\;{\text{m}}^{-1}$ (thick medium) for a numerical aperture (NA) ${\mathrm{NA}}_{\mathrm{inc}}=1.0$ of the incident light. Calculating a near-field solution of Maxwell’s equations for one incident plane wave of the finer sampled thin medium takes 80 minutes on average, and about 18 minutes for the coarser sampled thicker medium. By fixing the polarization (out-of-plane) and amplitude of the incident plane waves to a constant value, the phase remains as the only degree of freedom for optimization.

In order to minimize the reflected light intensity, the far-fields are needed. These are calculated by means of a near-to-far-field transformation [30,31]. The integration contour is located slightly above the first discs when viewed from the detection direction. To provide a sufficiently fine angular resolution, the far-fields are evaluated at 4001 sampling points.

### 2.2. Reflection minimization via phase optimization

To enhance the energy density within a turbid medium, we minimize the reflected far-field intensity through iterative, feedback-based phase optimization. This minimization is performed using a partitioning algorithm [3,4]. The optimization relies on the far-field contributions from each incident plane wave within the ASPW. The optimization workflow operates as follows.

First, to initialize the optimization, each incident plane wave of the ASPW is assigned a random phase from a uniform distribution between −π and π .

Second, the algorithm randomly divides the incident waves into two equal-sized subsets. One subset remains static, acting as a reference background, while the phases of the second subset are modulated.

Third, a global phase shift is applied to the entire modulated subset. This shift is swept across 256 discrete steps (from 0 to 2π ), matching the 8 -bit resolution of a standard spatial light modulator.

Fourth, for each of the 256 steps, the algorithm evaluates the feedback signal, which is defined as the total reflected far-field intensity, integrated over the detection numerical aperture ( ${\mathrm{NA}}_{\det}$ ). For each incident plane wave specified by $k_{y}$ that is encompassed by ${\mathrm{NA}}_{\mathrm{inc}}$ , the reflected far-field in the direction given by the angle θ to the detector-facing normal is given by $E_{ref}(k_{y},\theta )$ . Consequently, the complex field at each angle θ is given by the coherent sum of the contributions of all N incident plane waves. The intensity at that angle is then given by the absolute square of the sum. The cost function C , which serves as the optimization objective, is defined as the integral of these intensities over all angles within the detection range of ${\mathrm{NA}}_{\det}$ :

(3) $$C=\int_{-\arcsin({\mathrm{NA}}_{\det})}^{\arcsin({\mathrm{NA}}_{\det})}{\left|\sum_{\left|k_{y}\right|\leq k_{\max,\mathrm{inc}}}E_{\mathrm{ref}}(k_{y},\theta )\right|}^{2}d\theta$$

where $k_{\max,\mathrm{inc}}=k_{0}\cdot {\mathrm{NA}}_{\mathrm{inc}}$ with $k_{0}=2\pi /\lambda$ dictates the maximum accessible wave-vector y component for the chosen illumination aperture. The absolute value $\left|k_{y}\right|$ symmetrically accounts for both positive and negative incident angles relative to the surface normal.

In the fifth and final step, the specific phase shift that yields the greatest reduction in the cost function is selected and permanently added to the modulated subset, thereby updating the global phase pattern.

Steps two through five constitute a single iteration. This process is repeated until the optimization converges on a stabilized minimum, which is determined empirically. Convergence was reached after 6000 iterations for the thin medium and 4000 iterations for the thick medium.

The results are averaged over 100 repetitions for the thin medium and 48 repetitions for the thick medium, each starting with a different randomly chosen initial phase pattern. We observed no significant difference in the converged results when varying the number of iterations or repetitions.

Following the optimization procedure, the resulting optimized phases for each plane wave of the ASPW are applied to their respective near-field solutions within the simulated medium. The superposition of the adjusted near-field solutions then yields the optimized internal field distribution. Based on the initial, unoptimized field distribution and the distribution after optimization, the unoptimized and optimized energy densities can then be calculated.

**Fig. 2.**
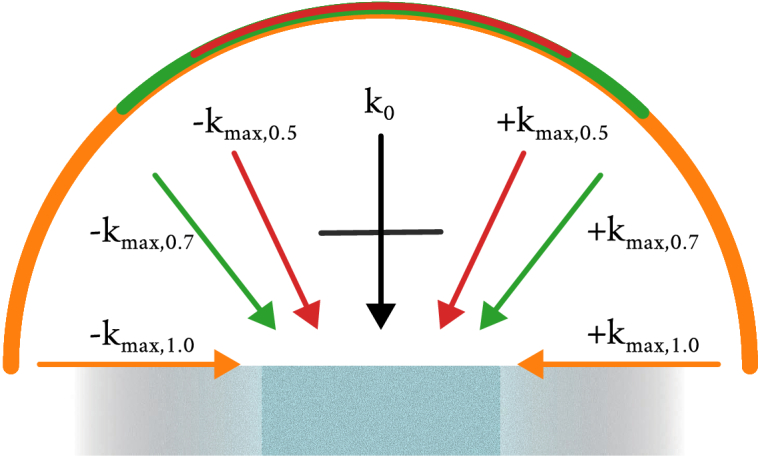
Schematic of the angular incidence and the detection ranges for varying numerical apertures. The central black arrow ( $k_{0}$ ) represents the incident plane wave normal to the scattering medium (blue). The colored arcs and corresponding arrows indicate the angular ranges for both light incidence and detection at NA=0.5 (red), NA=0.7 (green), and NA=1.0 (orange). The vectors marked $\pm k_{\max,\mathrm{inc}}$ represent the maximum accessible k-vectors for each respective aperture.

Simulations were conducted for numerical apertures of the incident light ${\mathrm{NA}}_{\mathrm{inc}}\in \{0.5,0.7,1.0\}$ , where lower values correspond to a reduced number N of contributing waves. Specifically, for the thin medium, N was set to 401 and 281 for ${\mathrm{NA}}_{\mathrm{inc}}=1.0$ and 0.7 , respectively. For the thick medium, N varied from 881 for ${\mathrm{NA}}_{\mathrm{inc}}=1.0$ to 617 for ${\mathrm{NA}}_{\mathrm{inc}}=0.7$ and 441 for ${\mathrm{NA}}_{\mathrm{inc}}=0.5$ . The detection aperture, determining the angular range for evaluating the far-field intensity during optimization, was similarly evaluated at ${\mathrm{NA}}_{\det}\in \{0.5,0.7,1.0\}$ . This allows for a direct comparison of the optimization scenario with conventional experiments where both irradiation and detection are carried out using the same lens. Figure 2 illustrates the angular illumination and detection ranges for numerical apertures of 0.5 (red), 0.7 (green), and 1.0 (orange). Light incident perpendicular to the scattering medium (blue) is denoted by $k_{0}$ , with the associated plane wave indicated by a horizontal line.

The choice of the partitioning algorithm over well-established alternatives, such as the stepwise and genetic algorithms cited in Refs. [3–7], is motivated by the feasibility of the presented method in potential experiments. The algorithm’s partitioning strategy maximizes the interference contrast during the optimization, facilitating rapid initial convergence and enhancing robustness against noise. This renders the algorithm superior, for example, to sequential methods, as demonstrated in the literature [3]. Sequential algorithms often require prior knowledge of the system under investigation, such as the decorrelation time, to achieve optimal performance. This is not the case for the partitioning algorithm [3], making it suitable for the presented approach, where no prior knowledge of the scattering medium is required. Furthermore, the partitioning algorithm has been shown to outperform both stepwise and genetic algorithms when optimizing the intensity across large areas [5].

## 3. Results

Simulations were performed for two configurations of the refractive index of the discs within the thin medium and one configuration for the thick medium. At first, the discs in the thin medium are solely configured as scattering objects, with an imaginary part of the refractive index equal to zero. Hereby, the reduced scattering coefficient, defined as $\mu_{s}^{\prime }=1/\ell^{\prime }=\mu_{s}\left(1-g\right)$ , resulted in $\mu_{s}^{\prime }=23.11\;{\text{mm}}^{-1}$ , where $\ell^{\prime }=43.26\;\mu \text{m}$ is the transport mean free path length, g=0.9016 is the anisotropy factor, and $\mu_{s}$ the scattering coefficient of the medium. Secondly, an imaginary part of 0.001 is assigned to the discs to introduce absorption. This results in a reduced scattering coefficient of $\mu_{s}^{\prime }=22.63\;{\text{mm}}^{-1}$ , a transport mean free path of $\ell^{\prime }=44.18\;\mu \text{m}$ , and an absorption coefficient of $\mu_{a}=1.63\;{\text{mm}}^{-1}$ , as well as g=0.9025 for the absorbing scenario of the thin medium. The optical parameters are derived from the analytical solution of Maxwell’s equations for a single homogeneous disc. This calculation incorporates the disc radius and total surface area, alongside the vacuum wavelength and the refractive indices of both the disc and the surrounding medium. Within this framework, the spatially resolved refractive index is defined as $n(x,y)=\sqrt{\epsilon (x,y)/\epsilon_{0}}$ , where ϵ(x,y) represents the local permittivity and $\epsilon_{0}$ is the vacuum permittivity.

The thick medium is designed with a reduced scattering coefficient of $\mu_{s}^{\prime }=23.06\;{\text{mm}}^{-1}$ and an absorption coefficient of $\mu_{a}=0.16\;{\text{mm}}^{-1}$ , as well as g=0.9017 and consequently $\ell^{\prime }=43.36\;\mu \text{m}$ . These parameters were chosen such that the ratio of $\mu_{a}$ to $\mu_{s}^{\prime }$ corresponds to that typically found in biological tissue in the red or infrared wavelength range. As $\mu_{a}$ is about two orders of magnitude smaller than $\mu_{s}^{\prime }$ , absorption is expected to play a subordinate role for this medium.

To facilitate comparison across different scattering regimes, the coordinates x and y , as shown in Fig. 1, are hereafter expressed as the dimensionless coordinates $x/\ell^{\prime }$ and $y/\ell^{\prime }$ normalized by the transport mean free path of the respective scattering medium.

### 3.1. Optimization of the energy density

Based on the optimized field distribution, previously described in Section 2.2, the time-averaged energy density is calculated as

(4) $$\langle u\rangle_{t}=\frac{1}{4}\left(\varepsilon (\vec{r})\vec{E}(\vec{r}){\vec{E}}^{*}(\vec{r})+\mu_{0}\vec{H}(\vec{r}){\vec{H}}^{*}(\vec{r})\right).$$

To ensure a rigorous comparison of the energy density across different numerical apertures and scattering media, we normalize the results obtained from Eq. (4) as follows. Each wave of the incident ASPW, and consequently its near and far-field contribution, is weighted using a Gaussian-distributed amplitude $A(k_{y})$ . This profile was selected because Gaussian beams are ubiquitous in laser-based microscopy. Hereby, the maximum amplitude is always located at the wave incident perpendicular to the surface. The full width at half maximum of the amplitude distribution is found at approximately ${\pm 38}^{\circ }$ for ${\mathrm{NA}}_{\mathrm{inc}}=1.0$ , at ${\pm 19}^{\circ }$ for ${\mathrm{NA}}_{\mathrm{inc}}=0.7$ , and at ${\pm 13}^{\circ }$ for ${\mathrm{NA}}_{\mathrm{inc}}=0.5$ .

To account for the varying number of angular components, we normalize the total incident power P to unity for every simulation according to:

(5) $$P=\sum_{n=1}^{N}{\left|A(k_{y,n})\right|}^{2}\frac{k_{x,n}}{k_{0}},$$

(6) $$k_{x,n}=\sqrt{k_{0}^{2}-k_{y,n}^{2}}.$$

Furthermore, all energy density values reported in this work are normalized relative to the energy density of an identical beam propagating in vacuum, which allows the results to be presented in absolute terms.

**Fig. 3.**
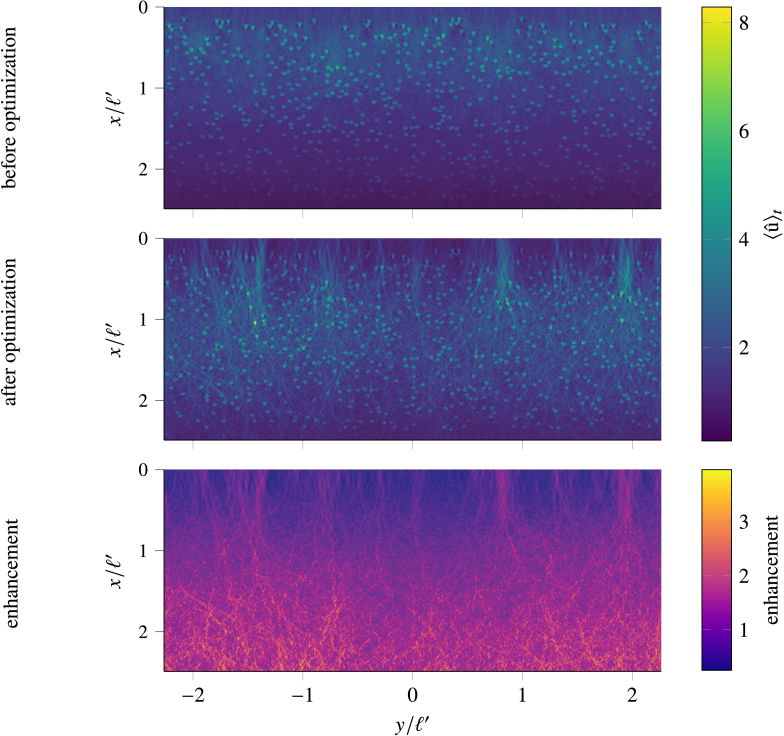
Normalized energy density for the thin absorbing medium with ${\mathrm{NA}}_{\mathrm{inc}}={\mathrm{NA}}_{\det}=1.0$ . Top: before optimization. Middle: after optimization. Bottom: enhancement factor. The energy density is normalized, in all scenarios, to the total incident power.

**Fig. 4.**
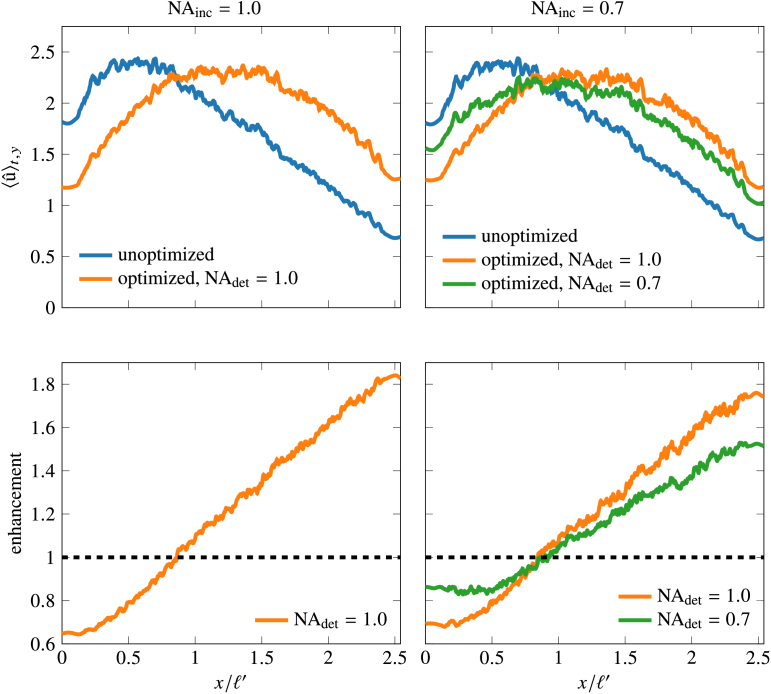
Depth-dependent energy density for the thin purely scattering medium. The top graphs show the normalized energy density averaged over the lateral coordinate. Blue curves indicate unoptimized illumination for the respective ${\mathrm{NA}}_{\mathrm{inc}}$ . The orange and green curves show the optimized energy density for the corresponding combinations of ${\mathrm{NA}}_{\mathrm{inc}}$ and ${\mathrm{NA}}_{\det}$ . The enhancements are shown in the bottom graphs.

**Fig. 5.**
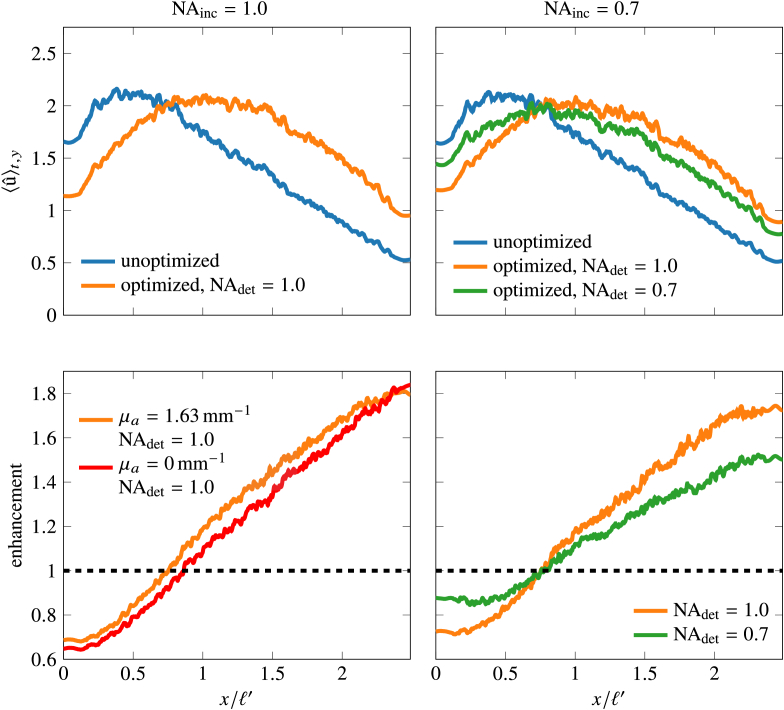
Depth-dependent energy density in the thin absorbing medium. The top graphs show the normalized energy density averaged over the lateral coordinate. Blue curves indicate unoptimized illumination for each ${\mathrm{NA}}_{\mathrm{inc}}$ . The orange and green curves show the optimized energy density for the corresponding combinations of ${\mathrm{NA}}_{\mathrm{inc}}$ and ${\mathrm{NA}}_{\det}$ . The enhancements are shown in the bottom graphs, with the curve for the purely scattering scenario added in red for comparison.

Figure 3 shows the simulation results for the normalized, spatially resolved energy density $\langle \hat{u}\rangle_{t}$ before the optimization (top), after the optimization (mid), and the spatially resolved enhancement (bottom). The results shown are the average of 100 individual repetitions for the thin medium with absorbing discs and ${\mathrm{NA}}_{\mathrm{inc}}={\mathrm{NA}}_{\det}=1.0$ . Each time with a different initial random phase pattern. Comparing the simulation results in the top and middle panels of Fig. 3, one realizes that minimizing the reflected far-field intensity using the aforementioned procedure leads to a redistribution into channels that transport light deep into the turbid medium. This preferential coupling of light into open transmission channels, as previously described, e.g., by Choi *et al.* [24], leads to an increase in the penetration depth and an enhancement of the energy density deep inside the medium. As can be seen in the bottom graph. To analyze the redistribution process of light penetrating the scattering media in the configurations with and without absorption, and the resulting enhancement of the energy density due to optimization, spatial averaging is performed along the y-axis. The obtained value of the energy density is denoted by $\langle \hat{u}\rangle_{t,y}$ . Figures 4 and 5 show $\langle \hat{u}\rangle_{t,y}$ , before and after optimization, for the thin medium with and without absorption. The energy density is plotted against depth for two configurations. One in which the medium contains purely scattering discs (see Fig. 4), and one in which the discs of the scattering medium also possess the aforementioned absorption properties (see Fig. 5). These are shown in the top two panels. The bottom graphs show the enhancement ratio between the optimized and unoptimized energy densities. The top two graphs in both figures show the unoptimized cases for ${\mathrm{NA}}_{\mathrm{inc}}=1.0$ (left) and ${\mathrm{NA}}_{\mathrm{inc}}=0.7$ (right) in blue. In the unoptimized case, the incident wavefront enters the scattering medium without phase control, which is why detection of the reflected intensity is not necessary, and therefore a specification for ${\mathrm{NA}}_{\det}$ is omitted in this case. The energy density initially increases before declining at greater depths. As noted in [32], this initial rise in the unoptimized scenario is driven by the transition of ballistic light, which decays exponentially according to the Beer–Lambert law, into diffuse scattered light. The accumulation of multiple scattering events creates a peak in energy density at a depth determined by the medium’s scattering coefficient and phase function. Additionally, front-surface reflections influence this initial distribution. Consequently, in the unoptimized case, most incident light is reflected or scattered before reaching deeper layers, and the interference between scattered paths is random.

However, if optimization redistributes and enhances the energy density inside the medium by adjusting the phase of the incident wavefront, this random interference can be controlled to some extent. Adjusting the incident wavefront so that reflected light ideally destructively interferes results in the energy being redistributed into open transmission channels inside the medium.

The resulting energy density distribution $\langle \hat{u}\rangle_{t,y}$ after optimization is shown in orange in the top left and right panel of Fig. 4 and Fig. 5 for ${\mathrm{NA}}_{\mathrm{inc}}={\mathrm{NA}}_{\det}=1.0$ (left) and ${\mathrm{NA}}_{\mathrm{inc}}=0.7$ and ${\mathrm{NA}}_{\det}=1.0$ (right). Furthermore, the right-hand graphs in each case show the result for ${\mathrm{NA}}_{\mathrm{inc}}={\mathrm{NA}}_{\det}=0.7$ in green.

In all cases, wavefront optimization consistently extends the depth at which high energy densities are sustained relative to the unoptimized case. Near the surface, optimization reduces energy density by suppressing reflection channels to favor transmission. This reduction is more pronounced at higher ${\mathrm{NA}}_{\mathrm{inc}}$ because the broader angular spectrum provides more degrees of freedom for effective reflection suppression. Consequently, while shallow-depth enhancement is lower for higher ${\mathrm{NA}}_{\mathrm{inc}}$ , constructive interference in the optimized transmission channels becomes dominant at greater depths, leading to a steady increase in enhancement (see Fig. 4 and Fig. 5, bottom panels). For comparison, the enhancement for ${\mathrm{NA}}_{\mathrm{inc}}={\mathrm{NA}}_{\det}=1.0$ of the solely scattering case of the thin medium is added to the bottom right graph of Fig. 5 depicted in red.

Both figures illustrate the transition from reflection channels at shallow depths to open transmission channels at greater depths, as shown by the enhancement curves that initially start below unity before rising significantly. As can be seen in the bottom left graph of Fig. 4 and Fig. 5, the enhancement exceeds a factor of 1.8 in the non-absorbing and absorbing case, resulting in almost double the energy density at a depth of $x/\ell^{\prime }\approx 2.3$ ( x≈100μm ), compared to the unoptimized case. This indicates that optimization has the strongest impact deeper in the medium, where multiple scattering would otherwise strongly suppress the light penetration.

From the right-hand graphs in both figures, it is clear that incomplete control of incident and detected light worsens total reflection suppression. Because this limits the energy redistributed into open transmission channels during phase optimization, any reduction in ${\mathrm{NA}}_{\mathrm{inc}}$ or ${\mathrm{NA}}_{\det}$ decreases the achievable enhancement (see Section 3.2).

As is evident from the top panels of Fig. 5, absorption attenuates the redistribution of light deep into the medium, resulting in a lower energy density compared to the non-absorbing case ( $\mu_{a}=0\;{\text{mm}}^{-1}$ ). Nevertheless, optimization using the full detection NA consistently yields a higher enhancement than partial detection (see bottom graphs). Notably, the orange enhancement curve is above the red curve because optimization proves more effective against the suppressed unoptimized baseline of the absorbing case. When wavefront shaping identifies constructive interference channels that couple efficiently into the medium, these specific modes experience less attenuation than the average paths under random illumination. While the purely scattering scenario (red curve) shows a steady linear increase of the enhancement with depth, the absorbing scenario (orange curve) seems to saturate, but is usually larger.

**Fig. 6.**
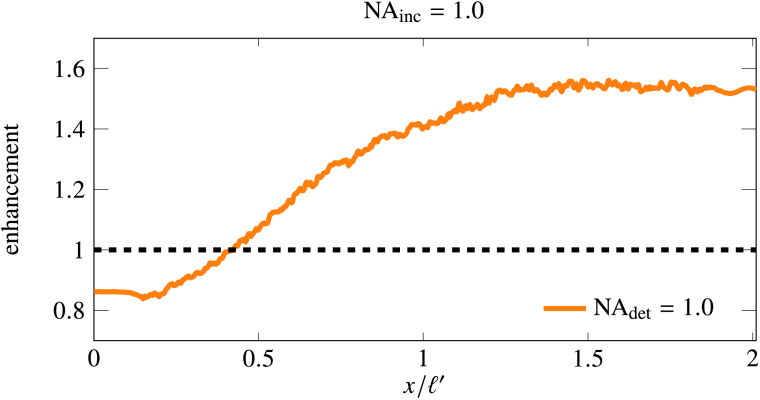
Depth-dependent, laterally averaged enhancement in the thin absorbing medium for $\mu_{s}^{\prime }=18.28\;{\text{mm}}^{-1}$ , $\ell^{\prime }=54.69\;\mu \text{m}$ , $\mu_{a}=14.61\;{\text{mm}}^{-1}$ , and ${\mathrm{NA}}_{\mathrm{inc}}={\mathrm{NA}}_{\det}=1.0$ .

**Fig. 7.**
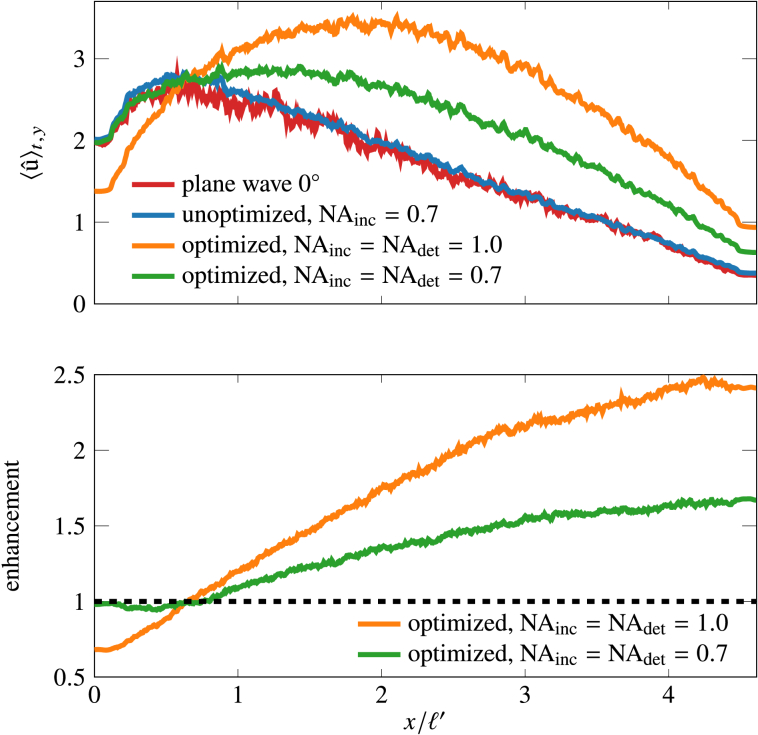
Depth-dependent energy density in the thick absorbing medium. Top: Normalized, laterally averaged energy density for a plane wave incident from $0^{\circ }$ (red), unoptimized illumination at ${\mathrm{NA}}_{\mathrm{inc}}=0.7$ (blue), optimized cases for ${\mathrm{NA}}_{\mathrm{inc}}={\mathrm{NA}}_{\det}=1.0$ (orange) and ${\mathrm{NA}}_{\mathrm{inc}}={\mathrm{NA}}_{\det}=0.7$ (green). Bottom: Enhancement curves.

At greater depths, absorption outweighs the energy density enhancement achievable through wavefront shaping, leading to saturation. This effect is exacerbated at a smaller ${\mathrm{NA}}_{\mathrm{inc}}=0.7$ and becomes even more pronounced as the absorption coefficient increases. Figure 6 depicts a configuration equivalent to the thin medium, modified only by changing the imaginary part of the refractive index to 0.01 . This changes the reduced scattering coefficient to $\mu_{s}^{\prime }=18.28\;{\text{mm}}^{-1}$ , where $\ell^{\prime }=54.69\;\mu \text{m}$ and the absorption coefficient to $\mu_{a}=14.61\;{\text{mm}}^{-1}$ , where g=0.9097 . In this instance, both ${\mathrm{NA}}_{\mathrm{inc}}$ and ${\mathrm{NA}}_{\det}$ are fixed at 1.0 to isolate the impact of the altered material properties. Due to the drastically increased absorption coefficient, the saturation mentioned above is very clearly evident. To further explore the saturation of the energy density at greater depths, we now investigate the thicker medium with the aforementioned optical properties (see Section 3). For this medium, the absorption coefficient is reduced by an order of magnitude compared to the absorbing case of the thinner medium. As the decreased attenuation allows the influence of wavefront shaping to persist further into the medium, saturation occurs at significantly larger depths, and is now mainly caused by diffuse light propagation. The obtained results are depicted in Fig. 7.

Regarding the unoptimized energy density, only a slight difference is observed between the two incident apertures. Due to power normalization, a larger proportion of the incident power originates from larger angles for ${\mathrm{NA}}_{\mathrm{inc}}=1.0$ compared to ${\mathrm{NA}}_{\mathrm{inc}}=0.7$ . As these angles propagate over longer path lengths on average, they are more likely to be absorbed at shallower depths within the medium. Furthermore, increasing the angular range of the incident light while maintaining the same incident power leads to increased reflection from the front surface. However, these differences are very small and vanish with increasing depth. For clarity, Fig. 7 hence only displays the unoptimized energy density for ${\mathrm{NA}}_{\mathrm{inc}}=0.7$ . It should also be noted that a higher numerical aperture results in a higher optimized energy density when the number and power of the incident waves are the same. This is because the larger NA expands the accessible k-space, enabling the excitation of high-frequency spatial modes and allowing for more precise interference control. This is also evident from simulations using the presented methodology.

Wavefront shaping of the light incident onto the thicker medium further increases the enhancement, reaching a maximum of about 2.5 for ${\mathrm{NA}}_{\mathrm{inc}}={\mathrm{NA}}_{\det}=1.0$ (orange) at a depth of $x/\ell^{\prime }\approx 4.38$ ( x≈190μm ). With apertures restricted to ${\mathrm{NA}}_{\mathrm{inc}}={\mathrm{NA}}_{\det}=0.7$ , the peak enhancement drops to approximately 1.7 (green). As a baseline, the normalized energy density for a plane wave incident from $0^{\circ }$ (red) is included, which closely follows the trend of the unoptimized energy density produced by random initial phase patterns.

For applications in medical therapeutics, the ratio of the maximum energy density to the energy density deep within the medium is important. In the unoptimized case shown in Fig. 7, this ratio is 7.53 . However, by optimizing the penetration depth, this ratio can be reduced to 3.64 for ${\mathrm{NA}}_{\mathrm{inc}}={\mathrm{NA}}_{\det}=1.0$ . The high ratio of the enhancement between large and shallow depths throughout the simulations indicates a notable surface-sparing effect. For ${\mathrm{NA}}_{\mathrm{inc}}={\mathrm{NA}}_{\det}=1.0$ in Fig. 7, it equals 3.65 . This is, for example, important if damage due to the comparatively high absorption of melanin at the skin surface is to be avoided.

**Fig. 8.**
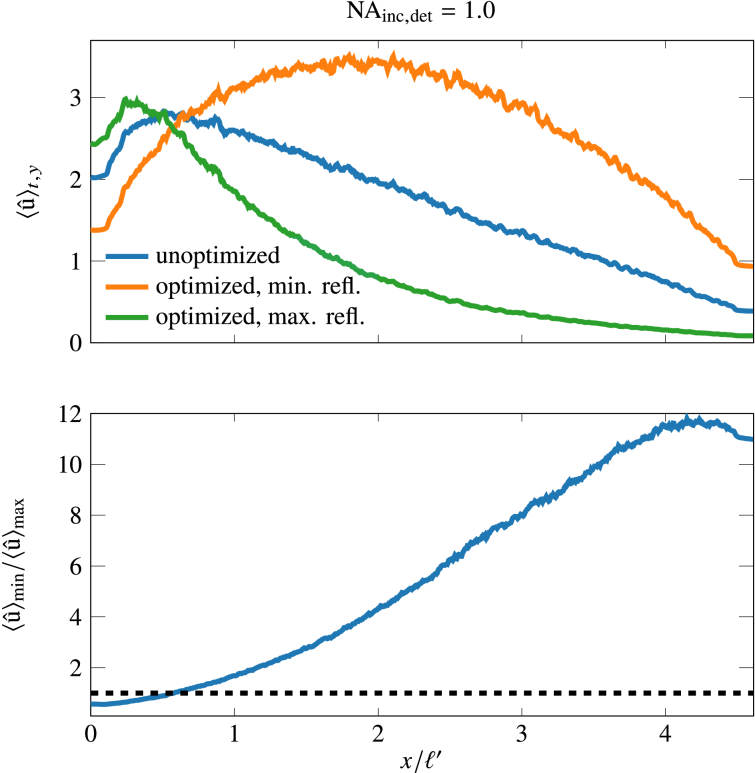
Depth-dependent energy density ${\mathrm{NA}}_{\mathrm{inc}}={\mathrm{NA}}_{\det}=1.0$ . Unoptimized illumination (blue), and minimized (orange), and maximized (green) reflected far-field intensity. Bottom: Enhancement ratio of the energy density for minimized reflection relative to the maximized reflection case.

The ability to manipulate light penetration is further demonstrated in Fig. 8. This figure contrasts the energy density profiles for wavefront shaping, where the feedback is not only the minimized (orange) but also the maximized (green) reflectance at ${\mathrm{NA}}_{\mathrm{inc}}={\mathrm{NA}}_{\det}=1.0$ . This comparison demonstrates that while minimizing the reflected far-field intensity facilitates a deeper redistribution of light, effectively delaying the transition to the diffuse regime, maximizing the reflected intensity has the opposite effect. In the latter case, destructive interference and the absorption present cause the energy density to decay rapidly with depth, significantly reducing penetration. Simultaneously, maximizing the reflection enhances the constructive interference within the reflection channels. As is evidenced by an increase in the energy density at shallow depths compared to both the unoptimized and the minimized-reflection cases.

By switching the optimization between these two extremes of minimal and maximal reflection or intermediate steps, it becomes possible to exert active control over the light field, effectively tuning the energy density at specific depths within the medium.

Figure 8 also shows the ratio of the laterally averaged, normalized energy densities for minimized and maximized reflection. At shallow depths, this ratio falls below unity, confirming that maximizing reflection concentrates light within the near-surface reflection channels through constructive interference. With increasing depth, the ratio rises sharply, where the depth-dependency can be used as a contrast mechanism for deep-tissue applications. Specifically, while the energy density at the superficial layers remains relatively stable, the optimization can deliver a twelvefold increase of the energy density at a depth of $x/\ell^{\prime }\approx 4.38$ ( x≈190μm ), when switching between states. This provides a high contrast mechanism for investigations and medical therapeutics. Based on our earlier observations regarding aperture limits, this ratio diminishes if the incident or detection NA is restricted.

### 3.2. Influence of a limited numerical aperture in detection

**Fig. 9.**
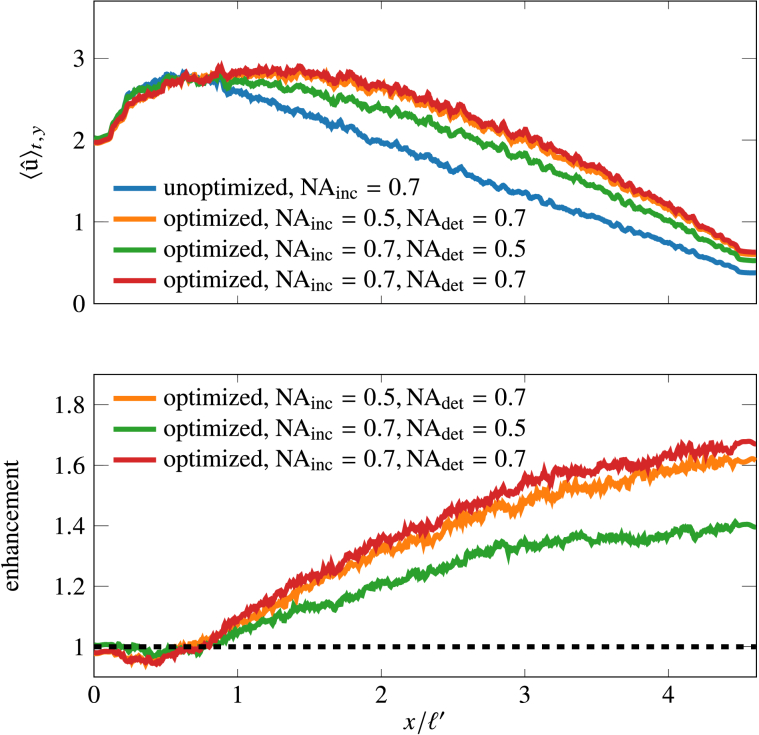
Depth-dependent energy density in the thick absorbing medium. Top: Normalized, laterally averaged energy density for unoptimized illumination with ${\mathrm{NA}}_{\mathrm{inc}}=0.7$ (blue) and optimized illumination with ${\mathrm{NA}}_{\mathrm{inc}},{\mathrm{NA}}_{\det}\in \{0.5,0.7\}$ (orange, green, red). Bottom: Enhancement curves.

As shown previously, limiting the illumination and detection NA has a strong influence in the form of diminished achievable enhancement. Notably, changing the detection NA has a greater influence than changing the illumination NA, as can be seen from the results in Fig. 9. The difference in the optimized energy density for ${\mathrm{NA}}_{\mathrm{inc}}=0.5$ and ${\mathrm{NA}}_{\det}=0.7$ compared to ${\mathrm{NA}}_{\mathrm{inc}}={\mathrm{NA}}_{\det}=0.7$ is smaller than for ${\mathrm{NA}}_{\mathrm{inc}}=0.7$ and ${\mathrm{NA}}_{\det}=0.5$ . Consequently, the difference in the achievable enhancement is also smaller. When the detection NA is limited, destructive interference is arranged only within the captured angular range, while constructive interference can still arise in undetected directions. Since backscattered light escaping into these undetected angles is not penalized by the cost function, the optimization can effectively reduce the feedback signal by redirecting light into this undetected region rather than redirecting it deeper into the medium. This suggests that prioritizing a high numerical aperture in detection is more critical for maximizing the penetration depth in practical optical systems, than prioritizing a high illumination NA.

Because of the limited angular detection range, undetected channels continue to carry significant reflection. This causes the optimized, laterally averaged energy density in Fig. 9 to remain similar in shape to the unoptimized case at shallow depths. It also explains that the enhancement for the green curves in Fig. 4, Fig. 5, and Fig. 7 is weaker.

**Fig. 10.**
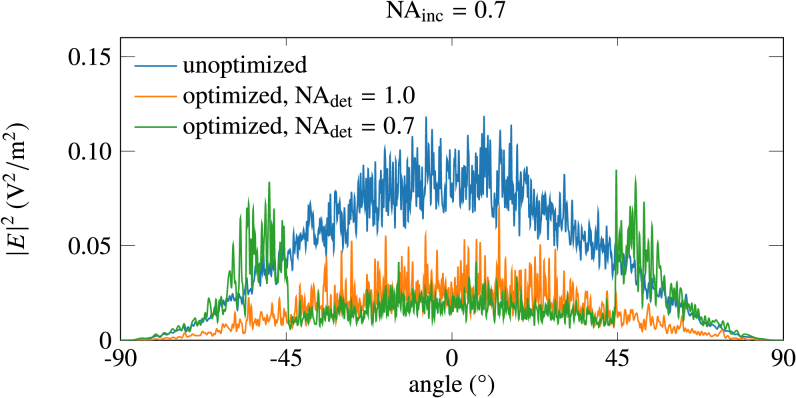
Reflected far-field intensity from the thin absorbing medium. Angular intensity for ${\mathrm{NA}}_{\mathrm{inc}}=0.7$ and various ${\mathrm{NA}}_{\det}$ , averaged over 100 random initial realizations. Unoptimized case (blue), optimized with full ${\mathrm{NA}}_{\det}=1.0$ (orange), and optimized for partial ${\mathrm{NA}}_{\det}=0.7$ (green).

A comparison of the reflected far-field intensity over the angular half-space in reflection (see Fig. 10) for the thin medium with purely scattering discs is shown, where ${\mathrm{NA}}_{\mathrm{inc}}=0.7$ . The unoptimized case is depicted in blue, the globally optimized reflected far-field with ${\mathrm{NA}}_{\det}=1.0$ in orange, and the result for partial angular detection, where ${\mathrm{NA}}_{\det}=0.7$ , in green. The results are averaged over 100 initial phase patterns. Local suppression within ${\mathrm{NA}}_{\det}=0.7$ appears more effective with partial detection, as the optimizer only needs to cancel a smaller subset of reflected channels. However, this results in more energy being shunted into an uncontrolled angular range. This leakage is evidenced by the increased far-field intensity of the green curve outside the targeted ${\mathrm{NA}}_{\det}$ , ultimately yielding a weaker global enhancement compared to the orange curve. Quantitatively, partial detection at ${\mathrm{NA}}_{\det}=0.7$ reduces the reflected intensity within the detected window by 78% relative to the unoptimized baseline. However, due to the redistribution of light into modes outside this detection range, the global reflected intensity is reduced by only 48% . By contrast, global minimization achieves 67% reduction of the total reflected intensity.

## 4. Conclusion

The presented results demonstrate that the energy density inside a two-dimensional turbid medium can be significantly enhanced by minimizing the reflected far-field intensity through phase optimization of the incident wavefront. The method presented can also be extended to three dimensions in a straightforward manner. However, this significantly increases the necessary computational resources to obtain the near-field solutions in the numeric investigation of the method. While this study utilizes a two-dimensional framework, the core physical mechanisms, specifically coherent reflection suppression and the more significant role of the detection NA compared to the illumination NA, translate fundamentally to experimental setups and investigations of real, three-dimensional scattering media. These media usually exhibit a refractive index difference to air, which causes specular reflections. Furthermore, the depolarization of light backscattered from deep within the bulk medium presents additional complexities. These points have to be addressed in three-dimensional experimental designs. When considering real tissue, the structure is more complex, compared to the simulated media in this work, as it can consist of several layers, and can be mesoscopically anisotropic and inhomogeneous. Non-static behavior such as blood flow or thermal drift results in a finite decorrelation time, necessitating high-speed optimization to maintain enhancement. Nevertheless, the choice of scattering and absorption properties makes the media under consideration suitable models to investigate the possibilities of influencing the energy density in tissue-like media by means of wavefront shaping.

Three simulation scenarios were investigated. First, the discs in the thin medium were assigned a real refractive index to create an absorption-free environment. In the second scenario, absorption was introduced to the discs of the thin medium to create a strongly absorbing regime. The third scenario involved scaling both the lateral extension and thickness of the medium and adjusting the refractive index of the discs to more closely mimic optical properties typically found within biological tissue in the red or infrared spectral range. Lowering the imaginary part in comparison to the second scenario, the scattering and absorption properties were adjusted to achieve a $\mu_{s}^{\prime }/\mu_{a}$ ratio of approximately two orders of magnitude. Typical values for the reduced scattering coefficient in biological tissue are in the order of $\mu_{s}^{\prime }\approx 1\;{\text{mm}}^{-1}$ . By using scaling principles of light transport in scattering media, the results shown above correspond to similar enhancements in biological tissue with a thickness of approximately 4mm .

In these simulations, both the incident and detection numerical apertures were evaluated at values of 0.5, 0.7, and 1.0, including various combinations of these settings. By preferentially coupling light into open transmission channels and suppressing reflection, wavefront shaping achieved an enhancement of the normalized average energy density of approximately 1.8 in the thin medium and 2.5 in the thick medium for ${\mathrm{NA}}_{\mathrm{inc}}={\mathrm{NA}}_{\det}=1.0$ . Hereby, absorption introduces a clear damping effect on the energy density profiles, most notably at greater depths, and consequently a diminished achievable enhancement. Comparative analysis with the purely scattering scenario seems to reveal that absorption induces an earlier saturation of the enhancement, as light is absorbed rather than being redistributed. The consistently high ratio of deep-tissue to surface enhancement indicates a robust surface-sparing effect, which is critical for enhancing the penetration depth while minimizing the risk of thermal damage to the skin.

Furthermore, in the thick medium, saturation occurs as the light enters the diffuse regime. Here, the influence of wavefront shaping on the incident phases is significantly diminished, as the number of available channels can no longer sustain a further increase in the penetration depth.

By shaping the incident wavefront to either minimize or maximize the reflected far-field intensity, we can selectively modulate constructive interference within the medium’s open transmission channels. Our data demonstrates a high depth discrimination: while the surface energy density remains relatively constant, we achieve a twelvefold increase in the energy density ratio between the minimized and maximized reflection states at greater depths. This spatial gating ensures that the energy density can be switched deep within the medium while superficial layers experience a smaller impact, providing a robust mechanism for targeted medical intervention.

Additionally, we showed that restricting the illumination to a subset of input channels reduces the achievable enhancement in the energy density. This is especially the case, when the detection is also limited to a subset of reflection channels. Restricting the detection aperture can lead to the redistribution of light into undetected angles, instead of redirecting it deeper into the medium. Consequently, the enhancement of the energy density is further decreased. Our results indicate that reducing the detection NA has a stronger influence on the achievable enhancement than reducing the illumination NA.

In summary, the findings suggest several possibilities to further improve the enhancement of the energy density. As demonstrated by the results obtained, absorption reduces the enhancement. Therefore, operating at a wavelength that minimizes the influence of absorption is generally advisable. Another straightforward approach is to increase the number of incident waves to increase the degrees of freedom within the optimization process. Extending the optimization by also modulating the amplitude of the incident light in addition to the phase allows the full complex field to be controlled, which is expected to further increase the enhancement. Moreover, it has to be mentioned that in the presented method, reducing the reflected light does not guarantee an increase in the energy density inside the scattering medium, when the detection aperture is restricted.

The simulation procedure presented and the resulting insights are relevant for a range of applications in which controlled light delivery into highly scattering media is essential. These include diffuse photodynamic therapy, blood vessel coagulation, and epilation.

## Data Availability

Data underlying the results presented in this paper are not publicly available at this time but may be obtained from the authors upon reasonable request.

## References

[1] Gigan S. Katz O. de Aguiar H. B. et al., “Roadmap on wavefront shaping and deep imaging in complex media,” Journal of Physics: Photonics 4, 042501 (2022). 10.1088/2515-7647/ac76f9

[2] Vellekoop I. M. Mosk A. P., “Focusing coherent light through opaque strongly scattering media,” Opt. Lett. 32(16), 2309–2311 (2007). 10.1364/OL.32.002309 17700768

[3] Vellekoop I. Mosk A., “Phase control algorithms for focusing light through turbid media,” Opt. Commun. 281(11), 3071–3080 (2008). 10.1016/j.optcom.2008.02.022

[4] Vellekoop I. M., “Feedback-based wavefront shaping,” Opt. Express 23(9), 12189–12206 (2015). 10.1364/OE.23.012189 25969306

[5] Ojambati O. S. Hosmer-Quint J. T. Gorter K.-J. et al., “Controlling the intensity of light in large areas at the interfaces of a scattering medium,” Phys. Rev. A 94(4), 043834 (2016). 10.1103/PhysRevA.94.043834

[6] Popoff S. M. Goetschy A. Liew S. F. et al., “Coherent control of total transmission of light through disordered media,” Phys. Rev. Lett. 112(13), 133903 (2014). 10.1103/PhysRevLett.112.133903 24745422

[7] Conkey D. B. Brown A. N. Caravaca-Aguirre A. M. et al., “Genetic algorithm optimization for focusing through turbid media in noisy environments,” Opt. Express 20(5), 4840–4849 (2012). 10.1364/OE.20.004840 22418290

[8] Choi W. Mosk A. P. Park Q.-H. et al., “Transmission eigenchannels in a disordered medium,” Phys. Rev. B 83(13), 134207 (2011). 10.1103/PhysRevB.83.134207

[9] Liew S. F. Cao H., “Modification of light transmission channels by inhomogeneous absorption in random media,” Opt. Express 23(9), 11043–11053 (2015). 10.1364/OE.23.011043 25969200

[10] Vellekoop I. M. Mosk A. P., “Universal optimal transmission of light through disordered materials,” Phys. Rev. Lett. 101(12), 120601 (2008). 10.1103/PhysRevLett.101.120601 18851352

[11] Kim M. Choi W. Yoon C. et al., “Exploring anti-reflection modes in disordered media,” Opt. Express 23(10), 12740–12749 (2015). 10.1364/OE.23.012740 26074528

[12] Popoff S. M. Lerosey G. Carminati R. et al., “Measuring the transmission matrix in optics: An approach to the study and control of light propagation in disordered media,” Phys. Rev. Lett. 104(10), 100601 (2010). 10.1103/PhysRevLett.104.100601 20366410

[13] Kim M. Choi W. Choi Y. et al., “Transmission matrix of a scattering medium and its applications in biophotonics,” Opt. Express 23(10), 12648–12668 (2015). 10.1364/OE.23.012648 26074520

[14] Kim M. Choi Y. Yoon C. et al., “Maximal energy transport through disordered media with the implementation of transmission eigenchannels,” Nat. Photonics 6(9), 581–585 (2012). 10.1038/nphoton.2012.159

[15] Davy M. Shi Z. Park J. et al., “Universal structure of transmission eigenchannels inside opaque media,” Nat. Commun. 6(1), 6893 (2015). 10.1038/ncomms7893 25892450 PMC4411295

[16] He Y. Wu D. Zhang R. et al., “Genetic-algorithm-assisted coherent enhancement absorption in scattering media by exploiting transmission and reflection matrices,” Opt. Express 29(13), 20353–20369 (2021). 10.1364/OE.426496 34266126

[17] Jeong S. Lee Y.-R. Choi W. et al., “Focusing of light energy inside a scattering medium by controlling the time-gated multiple light scattering,” Nat. Photonics 12(5), 277–283 (2018). 10.1038/s41566-018-0120-9

[18] Jeong S. Kim D.-Y. Lee Y.-R. et al., “Iterative optimization of time-gated reflectance for the efficient light energy delivery within scattering media,” Opt. Express 27(8), 10936–10945 (2019). 10.1364/OE.27.010936 31052946

[19] Kim D.-Y. Jeong S. Jang M. et al., “Time-gated iterative phase conjugation for efficient light energy delivery in scattering media,” Opt. Express 28(5), 7382–7391 (2020). 10.1364/OE.385557 32225968

[20] Lee Y.-R. Choi W. Jeong S. et al., “Wave propagation dynamics inside a complex scattering medium by the temporal control of backscattered waves,” Optica 10(5), 569–577 (2023). 10.1364/OPTICA.480154

[21] Bender N. Yamilov A. Goetschy A. et al., “Depth-targeted energy delivery deep inside scattering media,” Nat. Phys. 18(3), 309–315 (2022). 10.1038/s41567-021-01475-x

[22] Sarma R. Yamilov A. G. Petrenko S. et al., “Control of energy density inside a disordered medium by coupling to open or closed channels,” Phys. Rev. Lett. 117(8), 086803 (2016). 10.1103/PhysRevLett.117.086803 27588875

[23] Hong P. Ojambati O. S. Lagendijk A. et al., “Three-dimensional spatially resolved optical energy density enhanced by wavefront shaping,” Optica 5(7), 844–849 (2018). 10.1364/OPTICA.5.000844

[24] Choi W. Kim M. Kim D. et al., “Preferential coupling of an incident wave to reflection eigenchannels of disordered media,” Sci. Rep. 5(1), 11393 (2015). 10.1038/srep11393 26078088 PMC4650648

[25] Ott F. Fritzsche N. Kienle A., “Numerical simulation of phase-optimized light beams in two-dimensional scattering media,” J. Opt. Soc. Am. A 39(12), 2410–2421 (2022). 10.1364/JOSAA.474318 36520764

[26] Bender N. Goetschy A. Hsu C. W. et al., “Coherent enhancement of optical remission in diffusive media,” Proc. Natl. Acad. Sci. 119(41), e2207089119 (2022). 10.1073/pnas.2207089119 36191199 PMC9564826

[27] Osnabrugge G. Leedumrongwatthanakun S. Vellekoop I. M., “A convergent Born series for solving the inhomogeneous helmholtz equation in arbitrarily large media,” J. Comput. Phys. 322, 113–124 (2016). 10.1016/j.jcp.2016.06.034

[28] Krüger B. Brenner T. Kienle A., “Solution of the inhomogeneous maxwell’s equations using a born series,” Opt. Express 25(21), 25165–25182 (2017). 10.1364/OE.25.025165 29041187

[29] Taflove A. Hagness S. C., *Computational electrodynamics: the finite-difference time-domain method* (Artech House, Inc., Norwood, MA, 2000), 2nd ed.

[30] Schelkunoff S. A., “Some equivalence theorems of electromagnetics and their application to radiation problems,” Bell Syst. Tech. J. 15(1), 92–112 (1936). 10.1002/j.1538-7305.1936.tb00720.x

[31] Umashankar K. Taflove A., “A novel method to analyze electromagnetic scattering of complex objects,” IEEE Trans. Electromagn. Compat. EMC-24(4), 397–405 (1982). 10.1109/TEMC.1982.304054

[32] Ott F. Reitzle D. Krüger B. et al., “Distribution of light inside three-dimensional scattering slabs: Comparison of radiative transfer and electromagnetic theory,” J. Quant. Spectrosc. Radiat. Transfer 277, 107987 (2022). 10.1016/j.jqsrt.2021.107987

